# Epidemiology of tuberculous lymphadenitis in Africa: A systematic review and meta-analysis

**DOI:** 10.1371/journal.pone.0215647

**Published:** 2019-04-19

**Authors:** Daniel Mekonnen, Awoke Derbie, Andargachew Abeje, Abebe Shumet, Endalkachew Nibret, Fantahun Biadglegne, Abaineh Munshae, Kidist Bobosha, Liya Wassie, Stefan Berg, Abraham Aseffa

**Affiliations:** 1 Department of Medical Microbiology, Immunology and Parasitology, College of Medicine and Health Sciences, Bahir Dar University, Bahir Dar, Ethiopia; 2 Biotechnology Research Institute, Bahir Dar University, Bahir Dar, Ethiopia; 3 The Centre for Innovative Drug Development and Therapeutic Trials for Africa (CDT-Africa), Addis Ababa University, Addis Ababa, Ethiopia; 4 Geospatial Data and Technology Center, Bahir Dar University, Bahir Dar, Ethiopia; 5 Amhara Regional State Health Bureau, Felege Hiwot Referral Hospital, Bahir Dar, Ethiopia; 6 Department of Biology, Bahir Dar University, Bahir Dar, Ethiopia; 7 Armauer Hansen Research Institute, Addis Ababa, Ethiopia; 8 Animal and Plant Health Agency, Weybridge, the United Kingdom; Fundació Institut d’Investigació en Ciències de la Salut Germans Trias i Pujol, Universitat Autònoma de Barcelona, SPAIN

## Abstract

**Introduction:**

Tuberculous lymphadenitis is the most frequent form of extra-pulmonary TB (EPTB) and accounts for a considerable proportion of all EPTB cases. We conducted a systematic review of articles that described the epidemiological features of TBLN in Africa.

**Methods:**

Any article that characterized TBLN cases with respect to demographic, exposure and clinical features were included. Article search was restricted to African countries and those published in English language irrespective of publication year. The articles were retrieved from the electronic database of PubMed, Scopus, Cochrane library and Lens.org. Random effect pooled prevalence with 95% CI was computed based on Dersimonian and Laird method. To stabilize the variance, Freeman-Tukey double arcsine root transformation was done. The data were analyzed using Stata 14.

**Results:**

Of the total 833 articles retrieved, twenty-eight articles from 12 African countries fulfilled the eligibility criteria. A total of 6746 TBLN cases were identified. The majority of the cases, 4762 (70.6%) were from Ethiopia. Over 77% and 88% of identified TBLN were cervical in type and naïve to TB drugs. Among the total number of TBLN cases, 53% were female, 68% were in the age range of 15–44 years, 52% had a history of livestock exposure, 46% had a history of consuming raw milk/meat and 24% had history of BCG vaccination. The proportion of TBLN/HIV co-infection was much lower in Ethiopia (21%) than in other African countries (73%) and the overall African estimate (52%). Fever was recorded in 45%, night sweating in 55%, weight loss in 62% and cough for longer than two weeks in 32% of the TBLN cases.

**Conclusions:**

TBLN was more common in females than in males. The high prevalence of TBLN in Ethiopia did not show directional correlation with HIV. Population based prospective studies are warranted to better define the risk factors of TBLN in Africa.

## Introduction

Tuberculosis (TB) is one of the oldest chronic and complex infectious diseases and is caused by a group of bacteria belonging to the *Mycobacterium tuberculosis* complex (MTBC). The complex includes the human adapted species of *M*. *tuberculosis and M*. *africanum*, and zoonotic pathogens; *M*. *bovis*, *M*. *caprae*, *M*. *microti* and *M*. *pinnipedii* which affect cattle, goats/sheep, voles and seals/lions, respectively [1, 2]. The current body of evidence suggests that these mycobacteria might have co-evolved along with early hominids in East Africa since as far back as 3 million years ago [3, 4].

The 2017 WHO global TB report on Africa showed high TB mortality (41/100,000), incidence (254/100,000) and TB/HIV co-infection (34%) rates. Moreover, the treatment success rate was below the WHO target of ≥85% [5]. In 2016, as many as 82% and 85% of TB deaths were reported among HIV-negative and total TB patients, respectively, in Africa and WHO South East Asia Region [5–7]. Altogether, Africa is the worst affected region with TB.

On average in the world, pulmonary TB (PTB) accounted for 85% of the clinical forms of TB whereas extra-pulmonary TB (EPTB) accounted for the remaining 15% [5, 8]. The most common types of EPTB include TB of the lymphatics (TBLN), pleural, bone, meningeal, genitourinary and peritoneal TB [9–11]. However, the prevalence of EPTB and its predominant forms varies from country to country [10, 12–15]. For instance, Ethiopia reports an EPTB proportion of 32%; ranking third in case number globally next to India and Pakistan despite their much larger total population size [5] and this level has remained high over the years [10, 16, 17].

Global TB control efforts have largely ignored EPTB. This is because EPTB is generally considered non-infectious and as such inconsequential to the global epidemic [18]. However, recent data from northwest England have shown that the prevalence of active TB disease among household contacts of EPTB was high (440 per 100 000 contacts screened), indicating that EPTB cases might have substantial impact on TB transmission [19]. Moreover, it is conceivable that the slower annual decline rate of EPTB compared to PTB [11] could retard the progress towards the END-TB targets set by WHO [20, 21].

Among the risk factors studied for EPTB, immunological drivers are well known and have been comprehensively reviewed by O’Garra and colleagues [22]. Reports from USA and South Africa showed that race, sex and HIV are important risk factors for the development of EPTB [15, 23]. Another study from Brazil also described an association of EPTB with HIV and ethnicity [24]. On the other hand, Mehta *et al*. (1991) compared TB data between pre and post HIV era in the USA and concluded that EPTB was not associated with HIV there [25]. Berg *et al*. (2015) did not find any association of ethnicity, TB strain type or HIV co-infection with TBLN prevalence in Ethiopia [26]. Additionally, the role of other potential factors such as over diagnosis [27], bovine origin [28] or lineage tropism [29] were minimal and unable to explain the high incidence rate of TBLN in Ethiopia.

Several attempts have been made to investigate differences in infectivity and virulence of strains isolated from PTB and EPTB patients. Viedma *et al*. (2005) used an *ex vivo* competitive macrophage co-infection assay and a murine aerosol-infection model and reported that strains isolated from EPTB were more efficient and showed higher infectivity than strains derived from PTB sites. This report suggests a possible role for bacterial factors in determining the clinical phenotypes [30]. On the other hand, a study by Gomes *et al*. (2013) failed to detect any association between clinical phenotypes of TB and MTBC genotypes. Rather, they found a link between clinical phenotypes of TB and host factors [24].

Tuberculosis may be considered as a disease with a continuous and dynamic spectrum [31]; TBLN as one pole of the spectrum occurring with a relatively strong host resistance, and disseminated TB as the other pole with relatively weaker host resistance. TB lymphadenitis is distinct from disseminated TB where lymph nodes could be involved in addition to pulmonary illness. TBLN is a form of TB with no evidence of pulmonary involvement or TB illness in any other organ of the body. The same strain types have been isolated from TBLN and PTB cases in Ethiopia, suggesting that host and/or environmental factors might play a role in the pathogenesis of TBLN rather than strain tropism [29].

In general, pooled data on the epidemiology of TBLN in Africa were not available. Moreover, the factors behind the development of TBLN are not well understood and this might require a combined analysis of data from host (genomics, immunity, co-morbidity), environment and pathogen genomics and be triangulated using powerful statistical and mathematical tools [1].

## Objectives

The central thesis of this review was to determine the geo-spatial distribution of TB lymphadenitis in Africa and to characterize TBLN cases by different demographic (gender, age groups), exposure (previous TB treatment history, raw meat/ milk exposure, BCG vaccination) and clinical variables (HIV co-infection, fever, weight loss, night sweat, cough).

## Methods

### Protocol registration

This review protocol is registered at the National Institute for Health Research; PROSPERO international prospective register of systematic reviews with registration number CRD42018104170 at (https://www.crd.york.ac.uk/PROSPERO/#recordDetails).

### Eligibility criteria

Any article that characterized TBLN in African countries with respect to: gender, age, TBLN/HIV co-infection, lymph node features, exposure status (livestock, raw milk/meat, BCG vaccination and TB treatment) and cardinal TB symptoms was included. Peer review articles published in the English language irrespective of publication year were included. Tuberculous lymphadenitis cases diagnosed on clinical criteria plus cytology and/or bacteriology were included. Those, TBLN cases diagnosed on clinical criteria alone were excluded. Sample size was not used as inclusion or exclusion criterion.

### Information sources and search strategy

Articles were retrieved from the electronic data bases of PubMed, Scopus, Cochrane library, and Lens.org. The search was done using key words and MeSH term. The key words included tuberculosis lymphadenitis, tuberculous lymphadenitis, lymph node tuberculosis, and Africa. The full search was done by combining key words and related MeSH terms using Boolean operators. S1 Table shows the full search strategy.

### Study selection

All of the identified articles were imported to an Endnote library. Initial screenings were done by title followed by abstract and then full text reading. Articles were assessed independently for the fulfillment of the inclusion criteria by two authors (AD, AS). Disagreements regarding the inclusion or exclusion of articles were resolved by discussion.

### Data collection process and data items

Data from the selected articles were extracted by two authors (DM, AM) independently using excel data extraction sheet. Key indicators such as first author, year of publication, study period, country, number and types of TBLN cases, lymph node features, sex, age and TBLN/HIV co-infection status were extracted. Moreover, history of exposure to raw milk/meat, BCG vaccination, contact with chronic cougher, previous TB treatment history were also extracted. Furthermore, cardinal TB symptoms (fever, night sweat, weight loss, and cough for longer than two weeks) were extracted. Distribution of patients’ place of origin and number of TBLN cases were mapped using ArcGIS 10.3 (ArcGIS Desktop, ESRI 2011. Redlands, Canada).

### Risk of bias in individual studies

To assess risk of bias, two authors of this paper (DM, EN) independently used the seven item-based ROBINS-I risk of bias assessment tool [32]. Each item scored one point and discrepancies were resolved by a third independent author (FB). Moreover, to determine the certainty of evidence generated and strength of recommendations; Grading of *Recommendations Assessment*, *Development and Evaluation* (GRADE) tool was applied [33].

### Summary measures and synthesis of results

The collected data were analyzed using quantitative measures. For random effect meta-analysis, approximate likelihood approach was followed. Moreover, to make the normal distribution assumptions more applicable to significance testing and stabilize the variances; Freeman-Tukey double arcsine rooted transformation was done [34]. Furthermore, to estimate the transformed pooled prevalence, Dersimonian and Laird method was used [35]. Taken together, using the metan command in Stata, study estimate (ES) as prevalence was computed using Freeman-Tukey double arcsine root transformation with 95% confidence interval. In the forest plot, the box indicated weight of articles from random effect analysis. The crossed line is the 95% confidence interval (CI), the solid vertical line is zero to x-axis. The analysis was done using Stata 14 (Stata Corp. College Station, TX, US). Country/sub-region/ wise sub-group analysis was done with regard to TBLN types, TBLN features, gender, age and TBLN/HIV co-infection status. The summary measures were presented as forest plots and table.

### Risk of bias across studies

Statistical heterogeneity estimate among the articles estimate was assessed using Cochrane Q, I^2^ statistic and P-value. The I^2^ value of <25%, 25–50% and ≥ 50% was taken as low, moderate and high degree of heterogeneity, respectively [36]. To deal with heterogeneity, sub-group and sensitivity analyses were performed; possible publication bias was assessed using funnel plot asymmetry.

## Results

### Study selection

**Fig 1:**
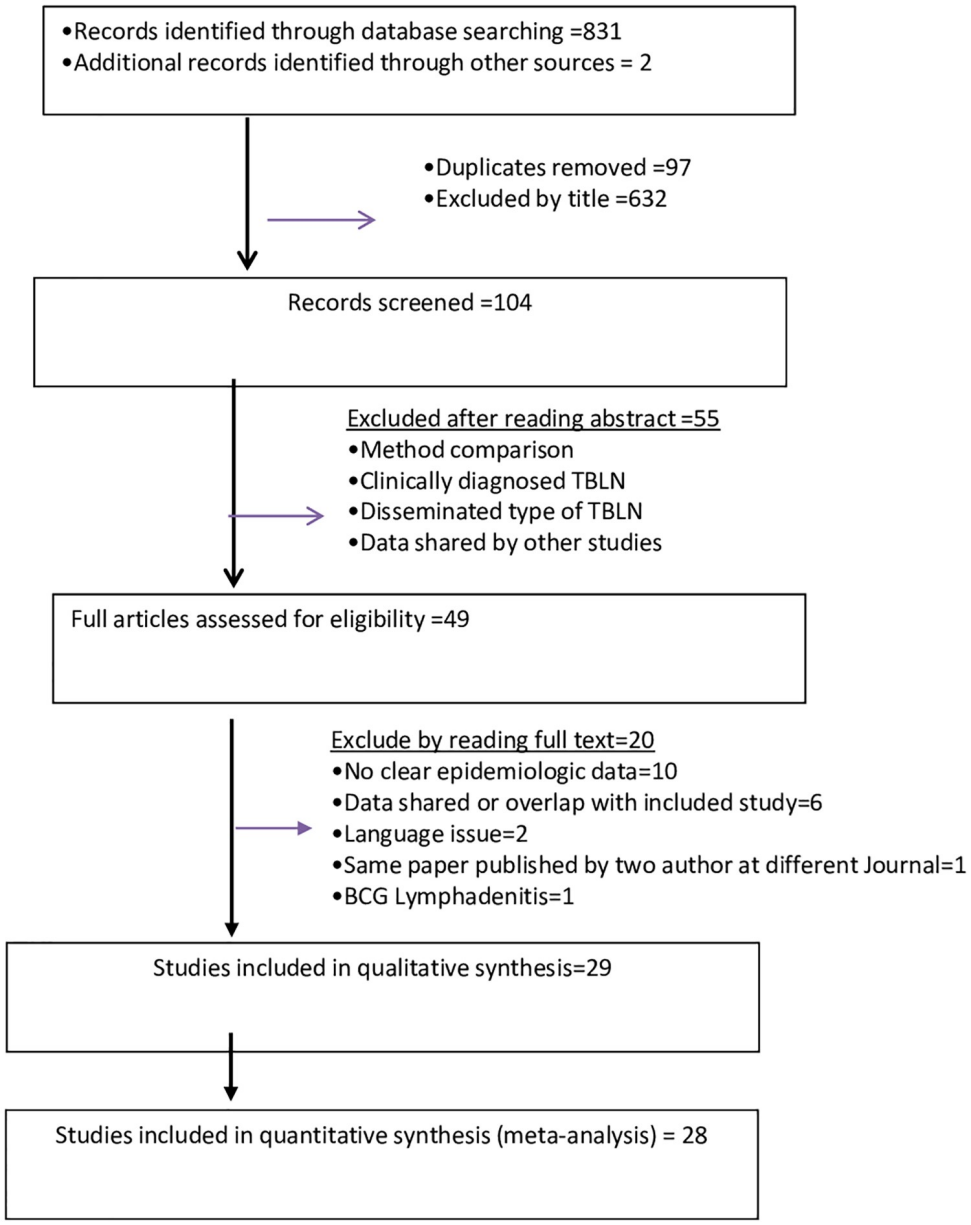
PRISMA flow diagram of literature selection, Africa, 2018.

A total of 831 articles were retrieved from the four electronic databases and imported to an Endnote library. Two additional articles were identified through hand searching in the Ethiopian Journal of Health Development and African Journals Online. After removing duplicates (97 articles), 736 articles were screened. Of these, 632 articles did not fulfill the inclusion criteria and they were removed. A further 55 articles were excluded for the same reason after reading the abstract. Twenty-eight articles were included in the quantitative analysis. Over all, full screening was done based on the preferred reporting items for systematic reviews and meta- analysis (PRISMA) flow diagram (Fig 1).

### Study characteristics

**Fig 2:**
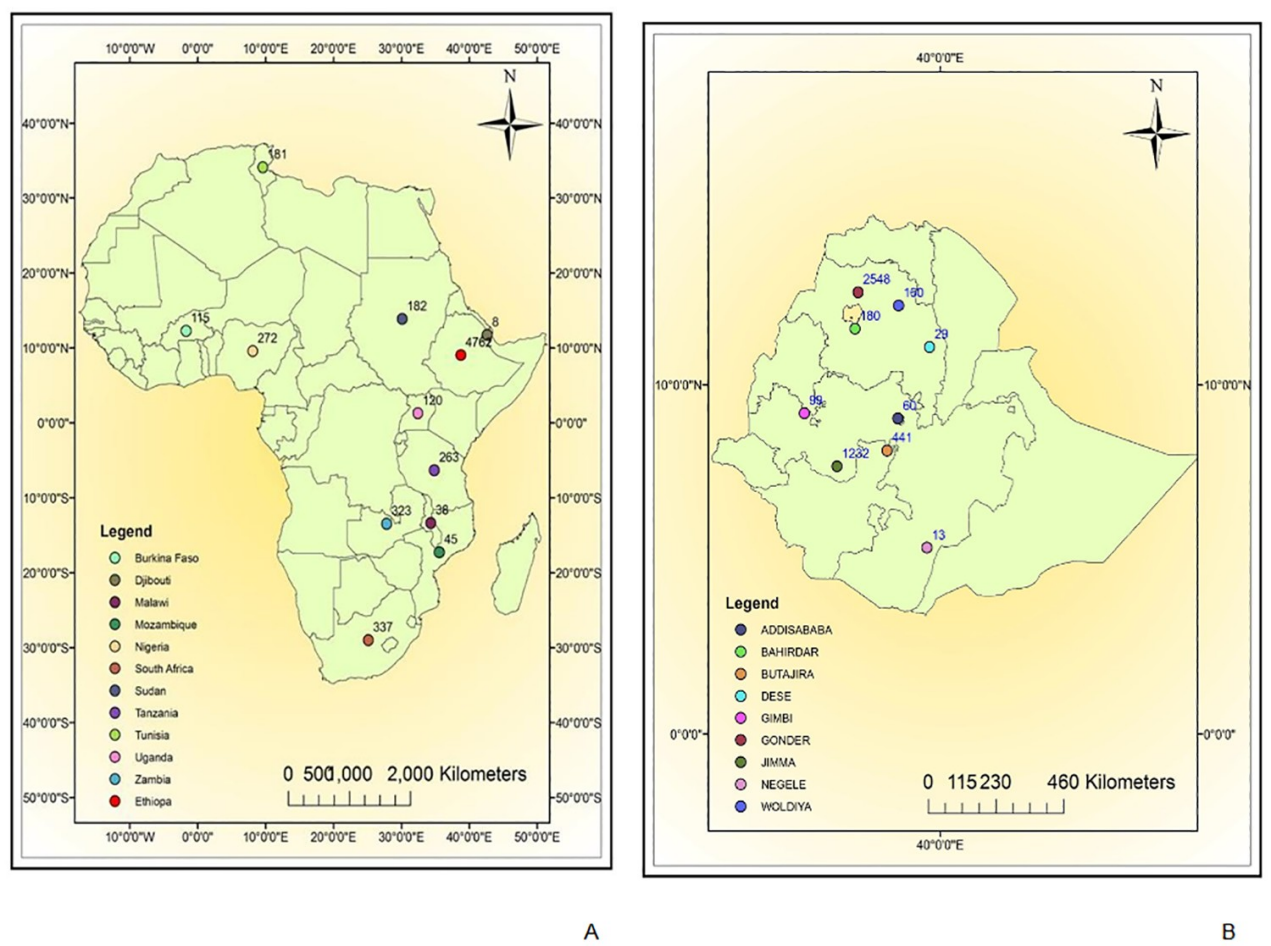
Geographic distribution and number of TBLN cases in Africa and Ethiopia, Africa, 1970–2015.

A total of 6746 TBLN cases from 12 African countries were reviewed. Majority of the cases, 4762 (70.6%) were from Ethiopia; Djibouti reported only eight confirmed TBLN cases. The geographic distribution of TBLN cases is summarized in Fig 2A and 2B. The spatial data used for the maps were taken from Map library which is a public domain that can be accessed at www.maplibrary.org.

Of the total of 28 articles reviewed, 14 articles were from Ethiopia [16, 26, 37–48] two articles each from Zambia [49, 50], South Africa [51, 52] and Nigeria [53, 54]. One article each were identified from Burkina Faso [55], Uganda [56], Djibouti [57], Mozambique [58], Sudan [59], Tunisia [60], Tanzania [61], and Malawi [62]. Data collection period of articles was between 1970 and 2015 (46 years) while the publication years range was between 1975 and 2018 (44 years) (Table 1).

**Table 1:** Reviewed articles and characteristics of TBLN cases, Africa, 1970–2015.

Author, year (reference)	Study period	Design/ Setting	Country	#TBLN pts	Sex		Age groups (Year)			HIV
					M	F	≤14	15–44	≥45	Po/N/U
Abdissa, 2014 [37]	2012	CS/H	Ethiopia	160	93	107	28	151	21	10/97/94
Abebe, 2012 [38]	2009	CS/C	Ethiopia	87	4	12	0	12	4	ND
Atomsa, 2014 [39]	2012	CCS/H	Ethiopia	48	18	30	0	47	1	0/48/0
Berg, 2015 [26]	2006–2010	CCS/C	Ethiopia	456	138	167	120	148	21	ND
Beyene, 2008 [40]	2005–2006	CS/C	Ethiopia	132	52	104	35	99	22	10/146/0
Bezabih, 2002 [41]	2000	CS/H	Ethiopia	128	72	56	27	90	11	ND
Bezabih, 2003 [42]	1999–2001	CS/H	Ethiopia	536	302	234	168	210	68	ND
Biadglegne, 2013 [16]	2012	CS/H	Ethiopia	225	95	130	26	171	29	ND
Fanosie, 2016 [43]	2015	CS/H	Ethiopia	87	74	67	29	76	36	24/12/0
Kidane, 2002 [44]	2000–2001	P/HC	Ethiopia	40	24	16	ND	ND	ND	11/29/0
Muluye, 2013 [45]	2003–2011	R/Lab	Ethiopia	2392	1098	1294	404	1689	299	ND
Tadesse, 2017 [46]	2013–2015	CS/HA	Ethiopia	304	143	161	33	166	105	24/232/7
Yassin, 2003 [47]	1998–2000	CS/HC	Ethiopia	107	51	56	ND	ND	ND	24/82/11
Zewdie, 2017 [48]	2014	CS/H	Ethiopia	60	30	30	0	52	8	ND
Bem, 1996 [49]	1981–1990	R/H	Zambia	238	247	216	53	182		115/11/342
Bem, 1997 [50]	1989–1990	P/H	Zambia	185	95	90	ND	ND	ND	157/28/0
Marais, 2006 [51]	2003–2004	P/HF	South Africa	35	69	89	35	0	0	ND
Khuzwayo, 2014 [52]	2007–2011	R/H	South Africa	302	196	162	ND	ND	ND	233/125/0
Onuigbo, 1975 [53]	1970–1974	R/ Lab	Nigeria	100	57	43	22	60	16	ND
Ukekwe, 2016 [54]	2000–2014	R/H	Nigeria	172	84	88	26	123	23	23/5/144
Beogo, 2013 [55]	2001–2010	R/H	Burkina Faso	115	53	62	16	74	21	42/72/0
Wamala, 2014 [56]	2010–2012	CS/H	Uganda	120	58	62	ND	ND	ND	75/39/7
Blouin, 2014 [57]	2010–2013	CS/HA	Djibouti	8	5	3	8	0	0	0/7/1
Viegas, 2015 [58]	2013–2014	CS.H	Mozambique	45	28	17	0	45	0	30/9/6
Ageep, 2012 [59]	2008–2011	R/Lab	Sudan	182	84	98	24	126	42	ND
Smaoui, 2015 [60]	2009–2013	R/ HA	Tunisia	181	65	116	ND	ND	ND	0/181/0
Richer, 1992 [61]	1984–1988	R/H	Tanzania	263	ND	ND	ND	ND	ND	ND
Boeree, 1998 [62]	1994–1995	CS/H	Malawi	38	ND	ND	0	30	8	32/6/0
Total				6746	3235	3510	1054	3551	735	

**CS:** Cross-sectional, **H:** Hospital, **CCS:** Comparative Cross-Sectional, **HA:** Hospital admitted patients, **C:** Community based study, **P:** Prospective study, **HC:** Health Center, **HF:** Health Facility, **R:** Retrospective/Registry review, **ND:** No data found, **#LNTB pts:** Number of Tuberculous lymphadenitis patients, **M:** Male, **F:** Female, **Po:** Positive, **N:** Negative, **U:** Unknown

Most articles contained complete and clear data about sex, age, TBLN/HIV co-infection status and types of TBLN (Table 1). However, some articles lacked complete information about livestock exposure, history of consumption of raw milk/meat/ and history of BCG vaccination. Different articles categorized age differently. Thus, best educated guess was applied to assign data to the respective age ranges. Groups with unknown HIV status were removed in the meta- analysis. Meta-analysis was done when at least two articles had the variables of interest.

### Risk of bias within studies

The risk of bias for each individual article was measured as no risk of bias, probably yes, yes and no information. Probably yes, yes and no information scored zero and no risk of bias got a score of one. The total score therefore ranged from zero to seven, with higher scores indicating higher quality of outcome. Of the total 28 articles reviewed; 17, 10 and one article showed an overall low, moderate and critical risk of bias, respectively (Table 2). Further, S2 Table shows that patient classification, measurement of outcome and reporting bias were the identified source of bias in the included articles. Overall, the included articles were judged as good quality.

**Table 2:** Robbins-I risk of bias summary for the included articles, Africa, 1970–2015.

Study, Year (Reference)	Overall quality score	Risk of bias judgement
Abdissa, 2014 [37]	4	Moderate
Abebe,2012 [38]	7	Low
Atomsa,2014 [39]	7	Low
Berg,2015 [26]	7	Low
Beyene, 2008 [40]	7	Low
Bezabih, 2002 [41]	7	Low
Bezabih, 2003 [42]	7	Low
Biadglegne, 2013 [16]	7	Low
Fanosie,2016 [43]	4	Moderate
Kidane, 2002 [44]	7	Low
Muluye, 2013 [45]	5	moderate
Tadesse,2017 [46]	7	Low
Yassin 2003 [47]	7	Low
Zewdie, 2018 [48]	7	Low
Bem, 1996 [49]	5	Moderate
Bem,1997 [50]	7	Low
Marais, 2006 [51]	4	Moderate
Khuzwayo, 2014 [52]	4	Moderate
Onuigbo,1975 [53]	7	Low
Ukekwe, 2016 [54]	7	Low
Beogo, 2013 [55]	4	Moderate
Wamala, 2014 [56]	5	Moderate
Blouin, 2014 [57]	5	Moderate
Viegas, 2015 [58]	7	Low
Ageep, 2012 [59]	7	Low
Smaoui,2015 [60]	7	Low
Richer,1992 [61]	5	Moderate
Boeree,1998 [62]	3	Critical

### Results of individual studies

**Fig 3:**
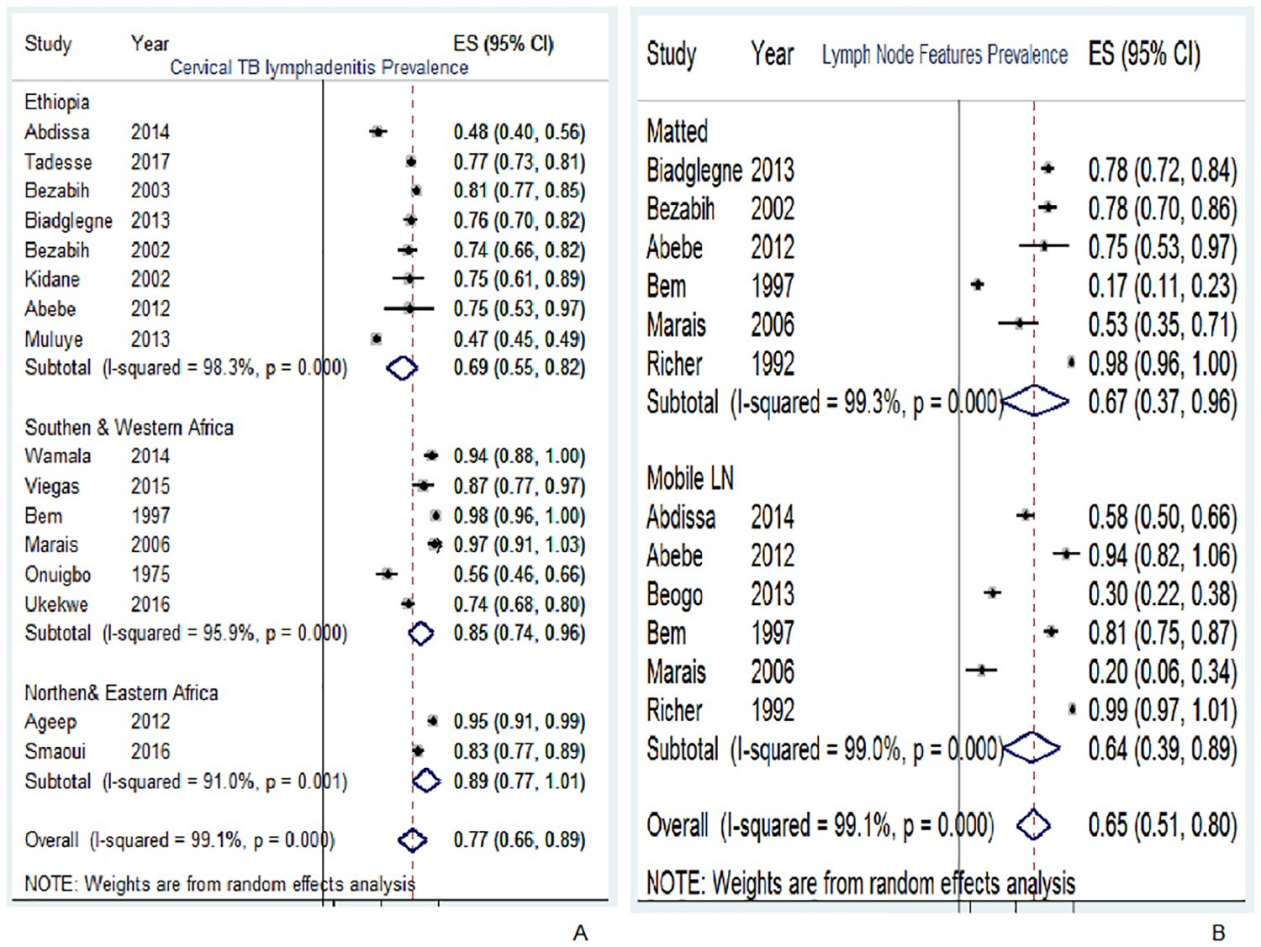
Pooled prevalence of TBLN types and features, Africa, 1970–2015.

**Fig 4:**
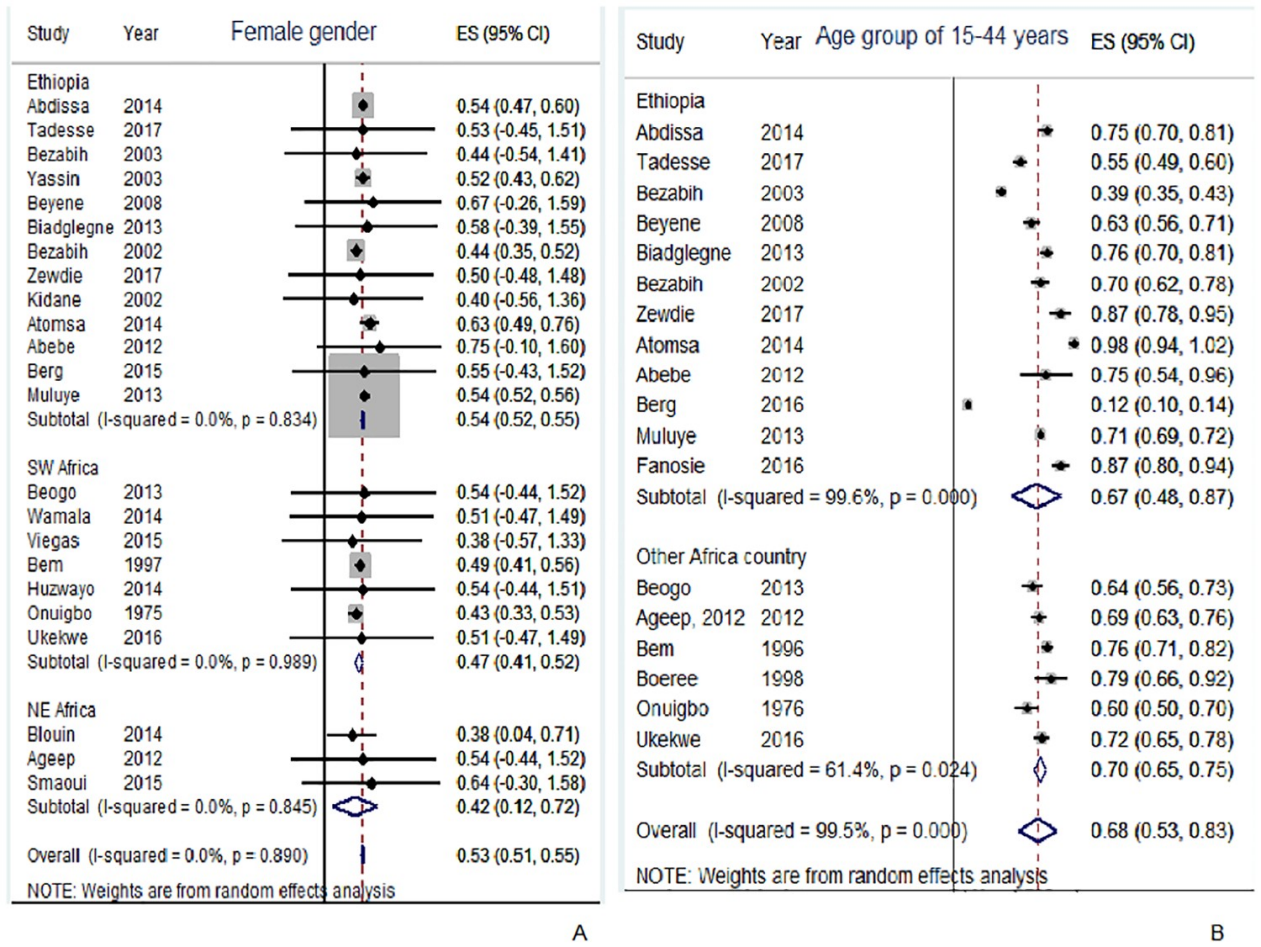
Proportion of female and age group of 15–44 years among TBLN cases, Africa 1970–2015.

**Fig 5:**
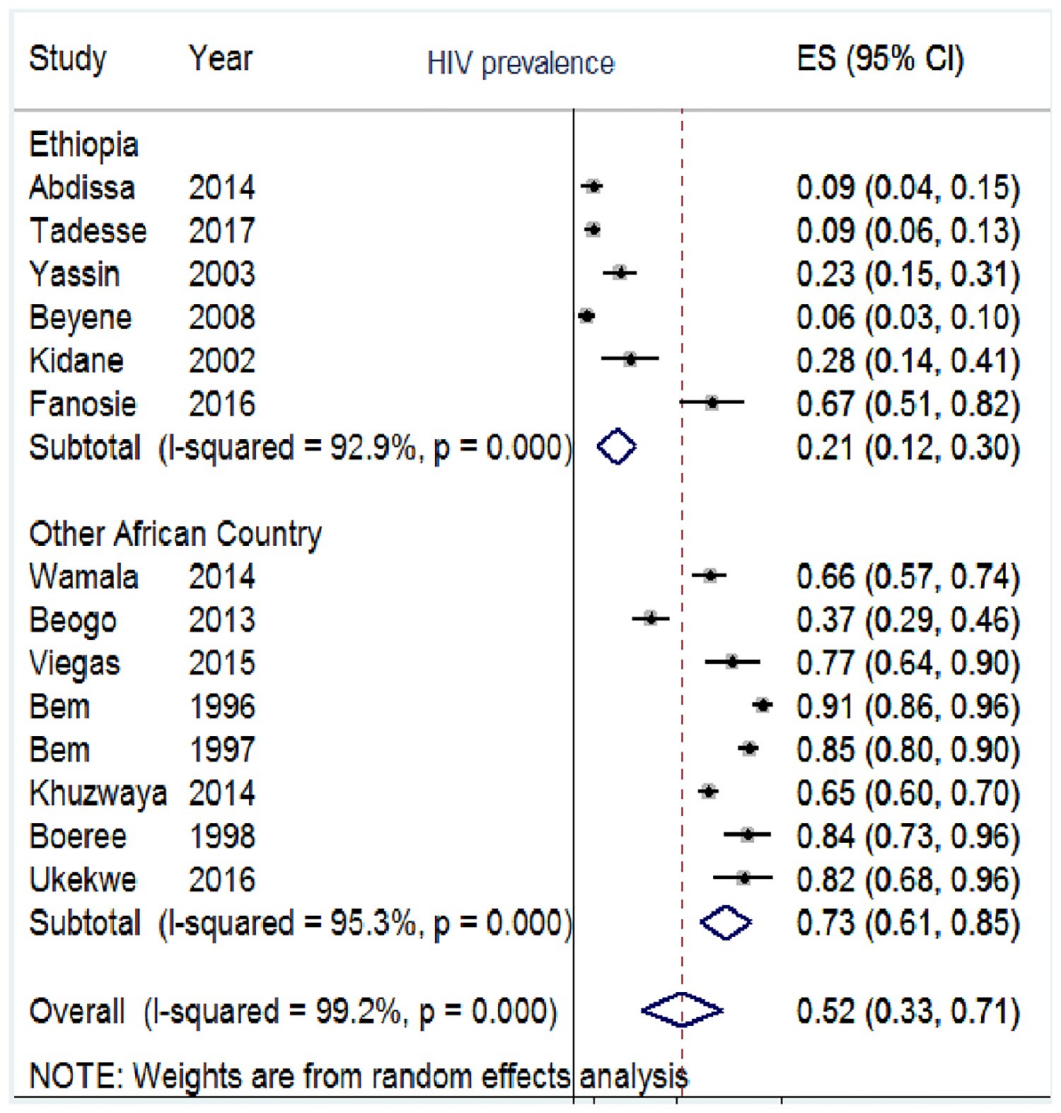
Proportion of TBLN/HIV co-infection among TBLN cases, Africa 1970–2015.

**Fig 6:**
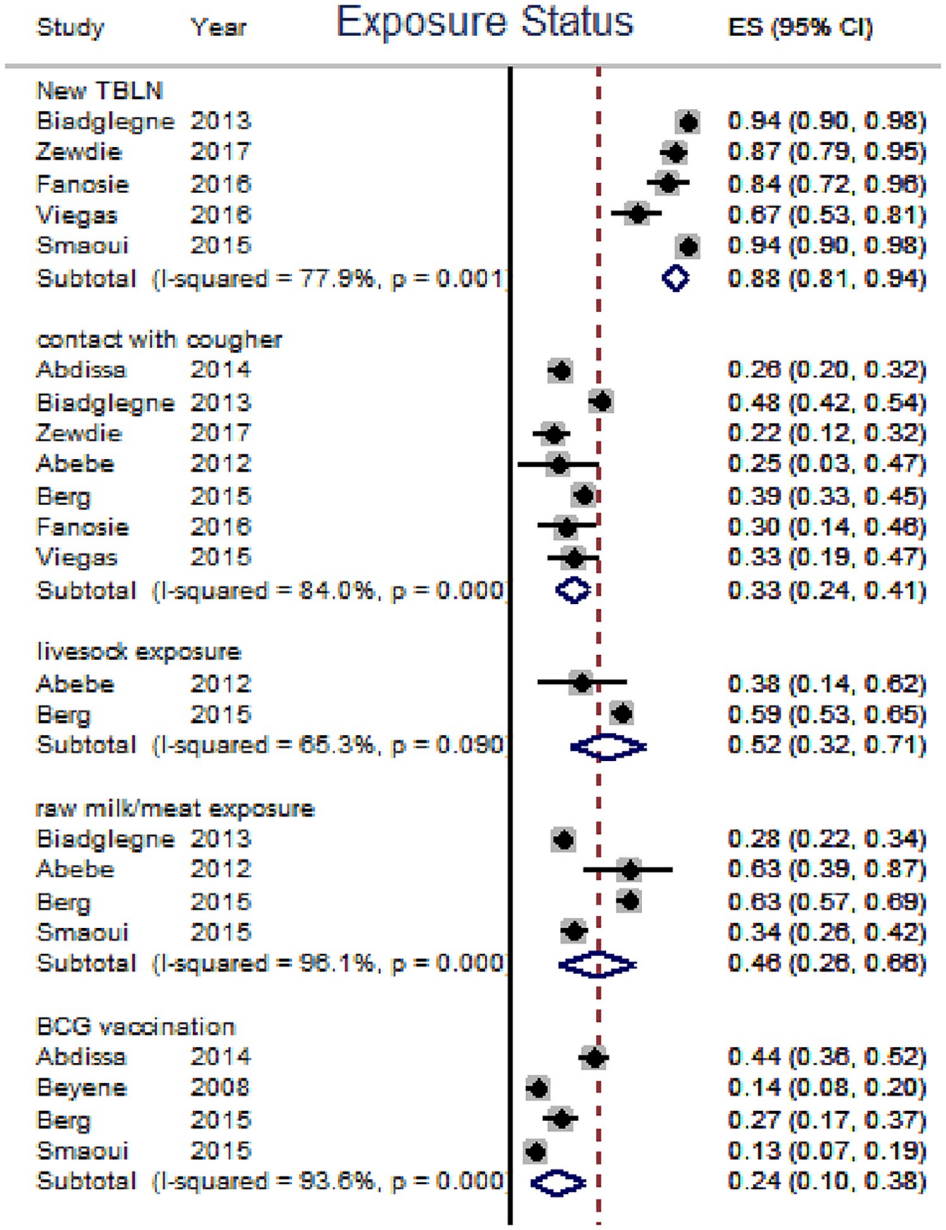
Prevalence of key exposure variables among TBLN cases, Africa, 1970–2015.

**Fig 7:**
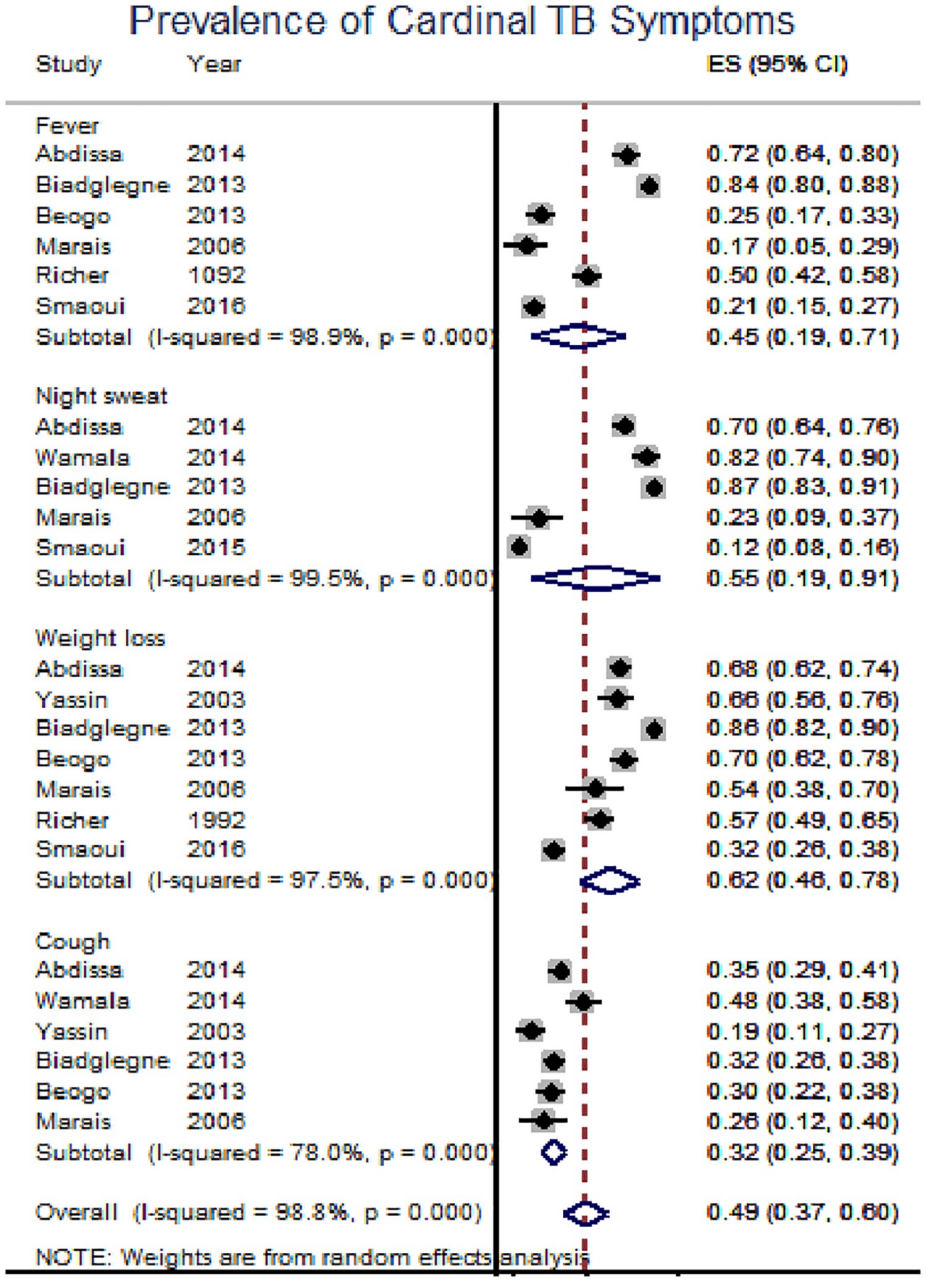
Prevalence of cardinal TB symptoms among TBLN cases, Africa, 1970–2015.

In this review, TBLN was disaggregated against 15 variables. The result of individual studies and its summary measure is presented using forest plot (Figs 3–7).

Cervical TBLN is the most prevalent form and ranged from 47% [45] to 98% [50]. The sub-group analysis showed that cervical LN type was lower in Ethiopia (69%) compared with articles from northern and eastern African countries (89%) and southern and western sub-regions (85%) (Fig 3A). On the other hand, Bem (1997) reported low prevalence of matted type TBLN,17% [50], while other studies reported higher than 50% prevalence of the matted types [16, 38, 41, 51, 61] (Fig 3B).

Three sub-group analyses were done on proportion of females among TBLN subjects; Ethiopia in one group, southern and western African articles in the second group and that of northern and other eastern African countries in a third group. Pooled prevalence of female gender among north and eastern African studies, excluding Ethiopia, reported the lowest prevalence (42%) followed by southern and western African countries (47%). In Ethiopia it was 54%. Individual article estimates of female proportion ranged from 38% to 75%. The pooled African female proportion was 53% (95%CI: 51–55%) showing more females with TBLN than males (Fig 4A). When sensitivity analysis was performed, Muluye *et al* [45] showed an influence on Ethiopian and African overall estimates. After removing the Muluye *et al* article, the female proportion turned out to be 52% in Ethiopia and 50% for overall Africa. We noted that Muluye *et al* [45] analyzed a large number of TBLN cases (Table 1) and the study quality was rated as good (Table 2). Part A of S1 Fig depicts the sensitivity analyses of articles included in gender wise meta- analysis.

Most of TBLN patients were in the age range of 15–44 years. Articles reported as low as 12% [26], 39% [42] and as high as 98% [39] prevalence of age range of 15–44 years among TBLN cases (Fig 4B). The sensitivity analysis in Part B of S1 Fig shows the influence of Berg *et al*. [26] and Muluye *et al*. [45] studies. Before removing these two studies, the pooled prevalence of age range of 15–44 years was 67%, 70% and 68% in Ethiopia, in other Africa countries and also in the overall African pooled estimate, respectively. However, when these two influential articles [29,45] were removed, the pooled prevalence turned out to be 72%, 70% and 72% in Ethiopia, in other African countries and in overall Africa, respectively.

The prevalence of HIV among TBLN cases showed a clear difference between Ethiopia and other African countries. The majority of cases in Africa other than Ethiopia showed higher prevalence of HIV among TBLN cases (Fig 5). The sensitivity analysis for TBLN/HIV co-infection showed minimal influence by a single study. Part C of S1 Fig depicts the sensitivity analysis.

The majority of TBLN cases were new which ranged from 67% [58] to 94% [16]). The individual and pooled prevalence of various exposure status are summarized in Fig 6. Moreover, Fig 7 shows that the prevalence of the cardinal TB symptoms lies between 32% and 62%) among TBLN cases.

### Synthesis of results

Seventy seven percent of TBLN cases included in this review were cervical lymph node (Fig 3A). Closer inspection of Fig 3B and Table 3 shows that matted and mobile type TBLN were more frequent than discrete and firm types, respectively. Moreover, TBLN was more frequent in female (53%) than in male patients (Fig 4A) and also more frequent in the age range of 15–44 years (68%) than in other age ranges (Fig 4B). The most surprising difference was the HIV prevalence among TBLN patients in Ethiopia, which differed significantly from the average in other African countries; 21% versus 73% (Fig 5).

**Table 3:** A summary of pooled prevalence to key variables among tuberculous lymphadenitis patients, Africa, 1970–2015.

Variables	#articles	PP	95%CI	I^2^%	QeE
Lymph node features					
Cervical	17	0.77	0.66–0.89	99	low
Matted	6	0.67	0.37–0.96	99.3	low
Mobile	6	0.64	0.39–0.89	99	low
Demographic and HIV profiles					
Female gender	23	0.53	0.51–0.55	0	low
15–44 years of age	18	0.68	0.53–0.83	99.5	low
TBLN/HIV coinfection-Ethiopia	6	0.21	0.12–0.3	92.9	low
TBLN/HIV co-infection-other African Countries	8	0.73	0.61–0.85	95.3	low
TBLN/HIV co-infection African overall	14	0.52	0.33–0.71	99.2	low
Exposure status					
New TBLN	5	0.88	0.81–0.94	77.9	low
Contact with Cougher	7	0.33	0.24–0.41	84	low
Livestock Exposure	2	0.52	0.32–0.71	65.3	low
History of eating raw meat/Drinking raw milk	4	0.46	0.26–0.66	96.1	low
BCG vaccination	4	0.24	0.1–0.38	93.6	low
Cardinal tuberculosis symptoms					
Fever	7	0.45	0.19–0.71	98.9	low
Night Sweating	5	0.55	0.19–0.91	99.5	low
Weight Loss	7	0.62	0.46–0.78	97.5	low
Cough >2 weeks	6	0.32	0.25–0.39	78	low

**HIV:** Human immune deficiency syndrome; **TBLN:** Tuberculous Lymphadenitis; **BCG:** Bacille of Calmitte and Guerin; **PP:** pooled prevalence, **CI:** Confidence interval; **QoE:** Quality of Evidence

The prevalence of exposure variables such as history of anti-TB drugs, history of contact with TB patients, BCG vaccination history, history of drinking raw milk and eating raw meat was generally lower than 50% and did not show any trend (Table 3 and Fig 6). Likewise, prevalence of cardinal TB symptoms among TBLN cases also varied. For example, the prevalence of weight loss and night sweating was 62% and 55%, respectively (Table 3 and Fig 7).

#### Risk of bias across studies

Except for female gender, the meta-analysis results were very heterogeneous and therefore, a random effect meta-analysis was done. The random effect analysis was also heterogeneous. To sort out the cause, publication bias was assessed. The funnel plot figures in S2 Fig shows the presence of possible publication bias. This bias might be due to missing of grey literatures across the continent and exclusion of non-English language articles. The other causes of heterogeneity might be due to differences in the recruitment criteria of TBLN cases and measurement of outcome variables. Sensitivity analysis was done for gender, age and TBLN/HIV co-infection for which over ten articles had been included. While omission of a single study at a time had minimal influence on the pooled prevalence of TBLN/HIV co-infection, it showed an influence on pooled prevalence of age groups [29, 45] and gender [45]. We noted that, the two influential articles have good methodological and outcome quality. Thus, it is less likely to influence the result away from the true pooled estimate. The GRADE pro system of grading the quality of evidence showed low quality of evidence. This is due to the methodological quality of the articles included in the review.

## Discussion

Tuberculous lymphadenitis (scrofula) has been recognized for thousands of years and remains one of the most common forms of EPTB [63]. Cervical TBLN is the most frequent form followed by axillary and inguinal TBLN. In the middle ages in Europe, it was believed that a touch from royalty could heal this disease [64]. Unlike PTB which is more common in males [5], our review identified a relatively higher percentage of females (53%) than males among TBLN cases (low quality of evidence) (Table 3 and Fig 4A). The link between being female and TBLN is not well known. However, reports have shown that differences in tumor necrosis factor, interleukin-10, CD4+ lymphocyte counts, endocrine, socioeconomic and cultural factors [64] might influence the development of TBLN. A review of 31 articles from Afghanistan, Pakistan, India and Bangladesh agrees with our report [18]. Katsnelson (2017) discussed pregnancy, diabetes, vitamin D deficiency and low protein consumption as potential factors associated with TBLN.

The pooled prevalence for the age group of 15–44 years was higher than for other age groups among TBLN cases (low quality of evidence). TBLN was previously considered a disease of childhood [65]. Recent reports also showed that high TBLN cases occur in the age range of 20 to 40 years [9, 64]. A critical review by Biadglegn *et al*. (2013) showed that EPTB (with TBLN being the most common presentation) was more common among young adults [17].

Sub-group analysis of TBLN/HIV co-infection by country/sub-region/ showed that the pooled prevalence of HIV among TBLN in African countries other than Ethiopia was 73% whereas it was 21% in Ethiopia (low quality of evidence). This indicates that Ethiopia’s high TBLN rate is probably unique in its epidemiology and seems to lack directional correlation with HIV. Multiple studies showed that HIV infection was significantly associated more with allopatric than sympatric host-pathogen relationships [66–68]. Absence of directional correlation between TBLN and HIV in Ethiopia might be a consequence of co-evolution. Moreover, evidence shows that EPTB is associated with HIV when it is the disseminated form rather than when it is exclusively localized [9]. The TBLN cases reviewed here are exclusively TBLN cases having no apparent pulmonary involvement. When considering Africa, the epidemiology of HIV among TBLN cases appears to be in line with other parts of the world in which HIV is the main driver of EPTB, including TBLN [69].

The majority (88%) of TBLN cases were newly identified cases showing no association between TBLN and TB treatment history. The history of eating raw meat/drinking raw milk among TBLN cases in Africa was 46% (low quality of evidence). In the past, it has been reported that 10–20% of all TBLN in Europe was caused by *M*. *bovis*, which was acquired from drinking unpasteurized milk [70]. However, recent studies from countries with similar settings (endemic bovine TB in cattle and no pasteurization) [71] have not shown such high prevalence of zoonotic TB. For instance, molecular analysis of 173 isolates from pastoral communities who had contact with livestock revealed as many as 160 *M*.*tuberculosis* and three *M*. *bovis*. Similarly, molecular analysis of 39 isolates from their camels, cattle and goats showed 24 *M*. *bovis* and 1 *M*. *tuberculosis* [72]. These data confirmed the low incidence of *M*. *bovis* as a cause for human TB. On the contrary, a systematic review of global epidemiology of TB due to *M*. *bovis* showed a higher rate (2.8%) among humans in Africa [73]. Such high prevalence of TB due to *M*. *bovis* is possibly because Müller *et al*. (2013) included articles reporting *M*. *bovis* using biochemical methods as diagnostic tool. It is known that biochemical method lacks specificity for identification of *M*. *bovis*. Taken together *M*. *bovis* was rarely detected in human TB [29, 74] but should not be ruled out as a zoonotic disease.

The prevalence of BCG vaccination history among TBLN cases in this review was 24%. However, the number of articles was a few (only 4 articles). Thus, the quality of the evidence is low; pending further investigation between BCG vaccination and TBLN. There are few reports about the effects of BCG on the incidence of TBLN except a report on its adverse effects among infants [75].

The prevalence of the cardinal TB symptoms among TBLN cases in Africa ranged from 32% with history of cough for longer than two weeks to 62% with record of weight loss. Overall, the prevalence of one or more systemic symptoms was 49%. Based on this report history of cough was less prevalent than weight loss. Unlike this study, a study from India showed that fever was the most prevalent symptom in TBLN [76]. Moreover, the prevalence of one or more systemic symptoms was 56.6% which is slightly higher than 49% in this report [76]. Another report from Turkey showed the prevalence of cough to be 26–33% which is in line with this report. However, night sweating of 29–36% which was reported by a study from Turkey [77] was lower than the present report (55%).

### Limitations

Due to methodological exclusion of articles published in languages other than English and missing of grey literatures; publication bias is likely high. Although we included a large number of articles in the review, each article however contained only few variables. Thus, prevalence estimate was based on a small sample size which might make our pooled estimate imprecise. In addition, most of the included articles were chart reviews and retrospective in nature likely introducing clinical and methodological heterogeneity. These collectively reduce the quality of the generated evidence.

### Conclusions

This review is the first comprehensive meta-analysis that estimated pooled prevalence for key demographic, exposure, and clinical variables that could characterize TBLN. Of the total 28 articles included in the review, 19 were from the Horn of Africa with most of these from Ethiopia (14 studies) suggesting more clustering of TBLN in Eastern Africa than in other sub regions. Within Ethiopia, TBLN was also relatively more clustered in agrarian than in pastoral regions.

Most TBLN (77%) were cervical in type, matted (67%) and mobile (64%) in their feature. The majority (68%) were in the age-range of 15–44 years. Unlike PTB which is more prevalent among males; TBLN is slightly higher among females (53%) and this requires further investigation. The TBLN/HIV co-infection rate was 52% in overall Africa, 21% in Ethiopia and 73% in the rest of Africa excluding Ethiopia which indicates the unique feature of TBLN epidemiology in Ethiopia. Eighty-eight percent of TBLN cases had no prior TB treatment history, 52% had livestock exposure and 24% had BCG vaccination scar. The prevalence of cardinal systemic symptoms among TBLN cases were 45%, 55%, 62% and 32% for fever, night sweating, weight loss, and history of cough for longer than two weeks, respectively.

To identify the most informative risk factors for TBLN, a meta-analysis and/or a prospective double population-based study is highly desirable. In addition, the host and pathogen genomic dimension and their evolutionary relationship should be investigated.

## Supporting information

S1 Table Literature search strategy. (DOCX)

S2 Table Full Robbins-I risk of bias assessment result. (XLSX)

S1 Fig Sensitivity analysis of gender, age and TBLN/HIV co-infection. (DOCX)

S2 Fig Funnel plots showing publication bias. (DOCX)

S1 File Completed PRISMA check list of the review. (DOCX)

## Abbreviations

BCG: Bacillus Calmette–Guerin
CI: Confidence Interval
EPTB: Extra-pulmonary TB
ES: Prevalence estimate of study
GRADE: Grading of *Recommendations Assessment*, *Development and Evaluation*
HIV: Human immune Deficiency Syndrome
I^2^: Inconsistency squared
MTBC: *Mycobacterium tuberculosis* complex
PP: pooled Prevalence
PRISMA: preferred reporting items for systematic reviews and meta- analysis
PTB: Pulmonary TB
QoE: Quality of Evidence
TB: tuberculosis
TBLN: Tuberculous lymphadenitis
WHO: World Health Organization
