# Supplementary material for: Epidemiology of tuberculous lymphadenitis in Africa: A systematic review and meta-analysis
Source: PLoS One. 2019 Apr 19;14(4):e0215647. doi: 10.1371/journal.pone.0215647 (PMC6474617; doi:10.1371/journal.pone.0215647)
Supplement: S1 Table — (DOCX) [file pone.0215647.s001.docx]

| Data Bases | Search Date | Search strategy | # articles |
| --- | --- | --- | --- |
| PubMed | 27March 2018 | ("Tuberculosis, Lymph Node"[Mesh]) AND "Africa"[Mesh] | 182 |
| Cochrane library | 27March 2018 | MeSH descriptor: [Tuberculosis, Lymph Node] explode all trees  Cochrane Central Register of Controlled Trials : Issue 3 of 12, March 2018 | 27 |
| Lens.org | 27March 2018 | abstract:(Tuberculous lymphadenitis) OR abstract:(tuberculosis lymphadenitis) OR abstract:(Lymph node tuberculosis)  Applied Filters: Publication Type = (exclude) unknown, journal article MeSH Heading = Humans, Tuberculosis, Lymph Node Keyword =tuberculosis, lymphadenitis | 100 |
| Scopus | 27 March 2018 | ( TITLE-ABS-KEY ( tuberculosis  AND lymphadenitis )  OR  TITLE-ABS-KEY ( tuberculous  AND lymphadentis )  OR  TITLE-ABS-KEY ( lymph  AND node  AND tuberculosis )  OR  TITLE-ABS-KEY ( lntb )  OR  TITLE-ABS-KEY ( lymph  AND node  AND tuberculous ) )  AND  DOCTYPE ( ar )  AND  ( LIMIT-TO ( AFFILCOUNTRY ,  "South Africa" )  OR  LIMIT-TO ( AFFILCOUNTRY ,  "Ethiopia" )  OR  LIMIT-TO ( AFFILCOUNTRY ,  "Tunisia" )  OR  LIMIT-TO ( AFFILCOUNTRY ,  "Nigeria" )  OR  LIMIT-TO ( AFFILCOUNTRY ,  "Morocco" )  OR  LIMIT-TO ( AFFILCOUNTRY ,  "Uganda" )  OR  LIMIT-TO ( AFFILCOUNTRY ,  "Egypt" )  OR  LIMIT-TO ( AFFILCOUNTRY ,  "Sudan" )  OR  LIMIT-TO ( AFFILCOUNTRY ,  "Tanzania" )  OR  LIMIT-TO ( AFFILCOUNTRY ,  "Zambia" )  OR  LIMIT-TO ( AFFILCOUNTRY ,  "Malawi" )  OR  LIMIT-TO ( AFFILCOUNTRY ,  "Cote d'Ivoire" )  OR  LIMIT-TO ( AFFILCOUNTRY ,  "Senegal" )  OR  LIMIT-TO ( AFFILCOUNTRY ,  "Cameroon" )  OR  LIMIT-TO ( AFFILCOUNTRY ,  "Congo" )  OR  LIMIT-TO ( AFFILCOUNTRY ,  "Kenya" )  OR  LIMIT-TO ( AFFILCOUNTRY ,  "Zimbabwe" )  OR  LIMIT-TO ( AFFILCOUNTRY ,  "Burkina Faso" )  OR  LIMIT-TO ( AFFILCOUNTRY ,  "Djibouti" )  OR  LIMIT-TO ( AFFILCOUNTRY ,  "Madagascar" )  OR  LIMIT-TO ( AFFILCOUNTRY ,  "Mozambique" )  OR  LIMIT-TO ( AFFILCOUNTRY ,  "Gabon" )  OR  LIMIT-TO ( AFFILCOUNTRY ,  "Ghana" )  OR  LIMIT-TO ( AFFILCOUNTRY ,  "Algeria" )  OR  LIMIT-TO ( AFFILCOUNTRY ,  "Botswana" )  OR  LIMIT-TO ( AFFILCOUNTRY ,  "Burundi" )  OR  LIMIT-TO ( AFFILCOUNTRY ,  "Central African Republic" )  OR  LIMIT-TO ( AFFILCOUNTRY ,  "Chad" )  OR  LIMIT-TO ( AFFILCOUNTRY ,  "French Guiana" )  OR  LIMIT-TO ( AFFILCOUNTRY ,  "Gambia" )  OR  LIMIT-TO ( AFFILCOUNTRY ,  "Mali" )  OR  LIMIT-TO ( AFFILCOUNTRY ,  "Rwanda" )  OR  LIMIT-TO ( AFFILCOUNTRY ,  "Angola" )  OR  LIMIT-TO ( AFFILCOUNTRY ,  "Benin" )  OR  LIMIT-TO ( AFFILCOUNTRY ,  "Guinea" )  OR  LIMIT-TO ( AFFILCOUNTRY ,  "Laos" )  OR  LIMIT-TO ( AFFILCOUNTRY ,  "Lesotho" )  OR  LIMIT-TO ( AFFILCOUNTRY ,  "Libyan Arab Jamahiriya" )  OR  LIMIT-TO ( AFFILCOUNTRY ,  "Niger" )  OR  LIMIT-TO ( AFFILCOUNTRY ,  "Yemen" ) )  AND  ( LIMIT-TO ( LANGUAGE ,  "English" ) )  AND  ( LIMIT-TO ( SRCTYPE ,  "j" ) ) | 522 |
