# Supplementary material for: Epidemiology of tuberculous lymphadenitis in Africa: A systematic review and meta-analysis
Source: PLoS One. 2019 Apr 19;14(4):e0215647. doi: 10.1371/journal.pone.0215647 (PMC6474617; doi:10.1371/journal.pone.0215647)
Supplement: S2 Fig — (DOCX) [file pone.0215647.s004.docx]

A

B

C

D

E
