# Supplementary material for: Epidemiology of tuberculous lymphadenitis in Africa: A systematic review and meta-analysis
Source: PLoS One. 2019 Apr 19;14(4):e0215647. doi: 10.1371/journal.pone.0215647 (PMC6474617; doi:10.1371/journal.pone.0215647)
Supplement: S1 File — (DOCX) [file pone.0215647.s005.docx]

| PRISMA Check list of the review | | | |
| --- | --- | --- | --- |
| Section/topic | # | Checklist item | Reported on page # |
| TITLE | | |  |
| Title | 1 | Epidemiology of Tuberculous Lymphadenitis in Africa: A systematic review and meta-analysis | 1 |
| ABSTRACT | | |  |
| Structured summary | 2 | **Introduction:** African is the most affected region in terms of tuberculosis (TB) morbidity, mortality and TB.HIV co-infection rates. The rather slow decline of TB in Africa can only be accelerated if the epidemiology of tuberculous lymphadenitis (TBLN) is better understood. Tuberculous lymphadenitis is the most frequent form of extra-pulmonary TB (EPTB) and accounts for a considerable proportion of all EPTB cases. Investigation into its epidemiology has however been neglected due to the high emphasis given to the infectious form of pulmonary TB (PTB). We conducted a systematic review of articles described the epidemiological feature of TBLN in Africa.  **Methods:** Any article that characterized TBLN cases with respect to demographic, exposure and clinical features were included. Articles search was restricted to African countries, published in the English language irrespective of publication year. The articles were retrieved from the electronic database of PubMed, Scopus, Cochrane library and Lens.org. Random effect pooled prevalence with 95% CI was computed based on Dersimonian and Laird method. To stabilize the variance, Freeman-Tukey double arcsine root transformation was done. The data were analyzed using Stata 14.  **Results:** Of the total 833 articles retrieved; twenty-eight articles from 12 African countries fulfilled the eligibility criteria. A total of 6746 TBLN cases were identified. The majority of the cases, 4762 (70.6%) were from Ethiopia. Over 77% and 88% of identified TBLN were cervical in type and naïve to TB drugs. Among the total TBLN cases, 53% were female, 68% were in the age range of 15-44 years, 52% had a history of livestock exposure, 46% had a history of consuming raw milk/meat and 24%hadBCG vaccination history. The proportion of TBLN/HIV co-infection was much lower in Ethiopia (21%) than in other African countries (73%) and regional estimate (52%). Fever was recorded in 45%, night sweating in 55%, weight loss in 62% and cough for longer than two weeks in 32% of the TBLN cases.  **Conclusions:** This review is the first comprehensive meta-analysis that generated pooled prevalence estimate of key demographic, exposure and clinical variables among TBLN cases. Hence, TBLN was more common among females and the high prevalence of TBLN in Ethiopia did not show directional correlation with HIV. A meta-analysis and/or double population based prospective study is warranted to better define the risk factors driving TBLN in Africa. | 2-3 |
| INTRODUCTION | | |  |
| Rationale | 3 | Tuberculosis may be considered disease with a continuous and dynamic spectrum [31] with TBLN as one pole of relatively strong host resistance and disseminated TB as the other pole of relatively weaker host resistance. TB lymphadenitis is distinct from disseminated TB where lymph nodes could be involved in addition to pulmonary illness. TBLN is a form of TB with no evident pulmonary involvement or TB illness in any other organ of the body. The same strains types have been isolated from TBLN as well as PTB cases in Ethiopia suggesting that host and/or environmental factors might play a role in the pathogenesis of TBLN rather than strain tropism [29].  In general, pooled data on the epidemiology of TBLN in Africa was not available. Moreover, the factors behind the development of TBLN are not well understood and this might require a combined data from host (genomics, immunity, co-morbidity), environment and pathogen genomics and be triangulated using powerful statistical and mathematical tools [1]. | 6 |
| Objectives | 4 | The central thesis of this review was to determine the geo-spatial distribution of TB lymphadenitis in Africa and to characterize TBLN cases by different demographic (gender, age groups), exposure (previous TB treatment history, raw meat/ milk exposure, BCG vaccination) and clinical variables (HIV co-infection, fever, weight loss, night sweat, cough). | 6 |
| METHODS | | |  |
| Protocol and registration | 5 | This review protocol is registered at National Institute for Health Research; PROSPERO international prospective register of systematic reviews with registration number CRD42018104170 at (https://www.crd.york.ac.uk/PROSPERO/#recordDetails). | 7 |
| Eligibility criteria | 6 | Any article that characterized TBLN in African countries with respect to: gender, age, TBLN/HIV co-infection, lymph node features, exposure status (livestock, raw milk/meat, BCG vaccination and TB treatment) and cardinal TB symptoms was included. Peer review articles published in English language irrespective of publication year were included. Tuberculous lymphadenitis cases diagnosed on clinical criteria plus cytology and/or bacteriology were included. Those, TBLN cases diagnosed by clinical criteria alone were excluded. Sample size was not used as inclusion or exclusion criteria. | 7 |
| Information sources | 7 | Articles have been retrieved from the electronic data base of PubMed, Scopus, Cochrane library, and Lens.org. | 7 |
| Search | 8 | The search was done using key words and MeSH term. The key words included tuberculosis lymphadenitis, tuberculous lymphadenitis, lymph node tuberculosis, and Africa. The full search was done by combining key words and related MeSH terms using Boolean operators. The table in S1 table shows the full search strategy.  Applied Filters: Publication Type = (exclude) unknown, journal article MeSH Heading = Humans, Tuberculosis, Lymph Node Keyword =tuberculosis, lymphadenitis | 7-8 |
| Study selection | 9 | All of the identified articles were imported to an Endnote library. Initial screenings were done by title followed by abstract and then full text reading. Articles were assessed independently for the fulfillment of the inclusion criteria by two authors (AD, AS). Disagreements regarding the inclusion or exclusion of articles were resolved by discussion. | 8 |
| Data collection process | 10 | Data from the selected articles were extracted by two authors (DM, AM) independently using excel data extraction sheet. | 8 |
| Data items | 11 | Key indicators such as first author, year of publication, study period, country, number and types of TBLN cases, lymph node features, sex, age and TBLN/HIV co-infection status were extracted. Moreover, history of exposure to raw milk/meat, BCG vaccination, contact with chronic cougher, previous TB treatment history were also extracted. Furthermore, cardinal TB symptoms (fever, night sweat, weight loss, and cough for longer than two weeks) were extracted. Distribution of patients’ place of origin and number of TBLN cases were mapped using ArcGIS 10.3 (ArcGIS Desktop, ESRI 2011. Redlands, Canada). | 8 |
| Risk of bias in individual studies | 12 | To assess risk of bias, two authors of this paper (DM, EN) independently used the seven item-based ROBINS-I risk of bias assessment tool [32]. Each item scored one point and discrepancies were resolved by a third independent author (FB). Moreover, to determine the certainty of evidence generated and strength of recommendations; Grading of Recommendations Assessment, Development and Evaluation (GRADE) tool was applied [33]. | 9 |
| Summary measures | 13 | Transformed pooled prevalence with 95%CI was the summary measure used. | 9 |
| Synthesis of results | 14 | The collected data were analyzed using quantitative measures. For random effect meta-analysis, approximate likelihood approach was followed. Moreover, to make the normal distribution assumptions more applicable to significance testing and stabilize the variances; Freeman-Tukey double arcsine rooted transformation was done [34]. Furthermore, to estimate the transformed pooled prevalence, Dersimonian and Laird method was used [35]. Taken together, using the metan command in Stata, study estimate (ES) as prevalence was computed using Freeman-Tukey double arcsine root transformation with 95% confidence interval. In the forest plot, the box indicated weight of articles from random effect analysis. The crossed line is the 95% confidence interval (CI), the solid vertical line is zero to x-axis. The analysis was done using Stata 14 (Stata Corp. College Station, TX, US). | 9 |

Page 1 of 2

| **Section/topic** | **#** | **Checklist item** | **Reported on page #** |
| --- | --- | --- | --- |
| Risk of bias across studies | 15 | Statistical heterogeneity among the articles estimate was assessed using Cochrane Q, I^2^ statistic and P-value. The I^2^ value of <25%, 25-50% and ≥ 50 % was taken as low, moderate and high degree of heterogeneity, respectively [36]. To deal with heterogeneity, sub-group and sensitivity analysis were performed; possible publication bias was assessed using funnel plot asymmetry. | 10 |
| Additional analyses | 16 | To deal with heterogeneity, sub-group and sensitivity analysis were performed; possible publication bias was assessed using funnel plot asymmetry. | 9 |
| **RESULTS** | | |  |
| Study selection | 17 | A total of 831 articles were retrieved from the four electronic databases and imported to an Endnote library. Two additional articles were identified through hand searching in the Ethiopian Journal of Health Development and African Journals Online. After removing duplicates (97 articles), 736 articles were screened. Of these, 632 articles did not fulfill the inclusion criteria and they were removed. A further 55 articles were excluded for the same reason after reading the abstract. Twenty-eight articles were included in the quantitative analysis. Over all, full screening was done based on the preferred reporting items for systematic reviews and meta- analysis (PRISMA) flow diagram (Fig1). | 10 |
| Study characteristics | 18 | A total of 6746 TBLN cases from 12 African countries were reviewed. Majority of the cases, 4762 (70.6%) were from Ethiopia; Djibouti reported only eight confirmed TBLN cases. The geographic distribution of TBLN cases are summarized in Fig 2A and 2B. The spatial data used for the maps were taken from Map library which is a public domain and can be accessed at www.maplibrary.org.  Of the total of 28 articles reviewed, 14 articles were from Ethiopia [16, 26, 37-48] two articles each from Zambia [49, 50], South Africa [51, 52] and Nigeria [53, 54]. One article each was from Burkina Faso [55], Uganda [56], Djibouti [57], Mozambique [58], Sudan [59], Tunisia [60], Tanzania [61], and Malawi [62] were identified. While the data collection period of articles lies between 1970 and 2015 (46 years); the publication years was between 1975 and 2018 (a range of 44 years) (Table 1).  Most articles contained complete and clear data about sex, age, TBLN/HIV co-infection status and types of TBLN (Table1). However, some articles lacked complete information about livestock exposure, history of consuming raw milk/meat/ and history of BCG vaccination. Different articles categorized age differently. Thus, best educated guess was applied to assign data to the respective age ranges. Groups with unknown HIV status were removed in the meta- analysis. Meta-analysis was done when at least two articles have the variables of interest. | 11-13 |
| Risk of bias within studies | 19 | The risk of bias for each individual article was measured as no risk of bias, probably yes, yes and no information. Probably yes, yes and no information scored zero and no risk of bias got a score of one. The total score therefore ranges from zero to seven, with higher scores indicating higher quality of outcome. Of the total 28 articles reviewed; 17, 10 and one article showed an overall low, moderate and critical risk of bias, respectively (Table 2). Further, table in S2 table shows that, patient classification, measurement of outcome and reporting bias were the identified source of bias in the included articles. Overall, the included articles judged as good quality. | 13-14 |
| Results of individual studies | 20 | In this review, TBLN was disaggregated against 15 variables. The result of individual studies and its summary measure is presented using forest plot (Fig 3-6).  Cervical TBLN is the most prevalent form and ranges from 47% [45] to 98% [50]. The sub-group analysis showed that cervical LN type was lower in Ethiopia (69%) compared with articles from northern and eastern African countries (89%) and southern and western sub-regions (85%) (Fig3A). On the other hand, while Bem (1997) reported low prevalence of matted type TBLN,17% [50]; other studies reported higher than 50% prevalence of the matted types [16, 38, 41, 51, 61] (Fig3B).  **Fig3: Pooled prevalence of TBLN types (A) and features (B), Africa, 1970-2015**  Three sub-group analyses were done on prevalence of female among TBLN subjects; Ethiopia in one group, southern and western African articles in the second group and that of northern and other eastern Africa country in a third group. Pooled prevalence of female gender among north and eastern African studies, excluding Ethiopia, reported the lowest prevalence (42%) followed by southern and western African countries (47%). In Ethiopia it was 54%. Individual article estimates of female prevalence ranged from 38% to 75%. The pooled regional female prevalence was 53% (95%CI: 51-55%) showing more female with TBLN than male (Figure 4a). When sensitivity analysis was performed Muluye *et al* [45] study showed an influence on Ethiopia and African overall estimate. The female proportion become 52% and 50% in Ethiopian and Africa overall, respectively. We noted that Muluye *et al* [45] study analyzed a large number TBLN cases (Table 1) and its quality was rated as good (Table 2). S1 Fig A depicts the sensitivity analysis of articles included in gender wise meta- analysis.  Most of TBLN patients were in the age range of 15-44 years. Articles reported as low as 12% [26],39% [42] to as high as 98% [39] prevalence of age range of 15-44 years among TBLN cases (Fig 4B). The sensitivity analysis in S1 Fig B showed the influence of Berg *et al*. [26] and Muluye *et al* [45] studies. Before removing these two studies, the pooled prevalence of age range of 15-44 years was 67%, 70% and 68% in Ethiopia, other Africa countries and overall African pooled estimate, respectively. However, when these two influential articles [29,45] were removed, the pooled prevalence became 72%, 70% and 72% in Ethiopia, other Africa countries and overall Africa, subsequently.  The prevalence of HIV among TBLN cases showed a clear difference between Ethiopia and other African countries. The majority of cases in Africa other than Ethiopia showed higher prevalence of HIV among TBLN cases (Fig4C). The sensitivity analysis for TBLN/HIV co-infection showed minimal influence by single study. S1 Fig C depicts the sensitivity analysis.  **Fig 4: TBLN cases by gender (A), age group (B) and HIV co-infection (C), Africa 1970- 2015**  The majority of TBLN cases were new (range: 67% [58] to 94% [16]). The individual and pooled prevalence of various exposure status are summarized in Fig 5. Moreover, Fig 6 shows that the prevalence of the cardinal TB symptoms (range:32%-62%) among TBLN cases.  **Fig5: Prevalence of key exposure variables among TBLN cases, Africa, 1970-2015**  **Fig6: Prevalence of cardinal TB symptoms among TBLN cases, Africa, 1970-2015** | 15-16 |
| Synthesis of results | 21 | Seventy-seven percent of TBLN included in this review were cervical lymph node (Fig3A). Closer inspection of figure 3B and table 3 shows that matted and mobile type TBLN were more frequent than discrete and firm types of lymph node, respectively. Moreover, TBLN was more frequent among females (53%) than among male patients (Fig4A) and also more frequent in the age range of 15-44 years (68%) than in other age ranges (Fig4B). The most surprising difference was the HIV prevalence among TBLN in Ethiopia, which differed significantly from the average in other African countries; 21% versus 73% (Fig 4C).  The prevalence of exposure variables such as history of anti-TB drugs, history of contact with TB patients, BCG vaccination history, history of drinking raw milk and eating raw meat was generally lower than 50% and did not show any trend (Fig5, Table 3). Likewise, prevalence of cardinal TB symptoms among TBLN cases also varied. For example, the prevalence of weight loss and night sweating were 62% and 55%, respectively (Fig6, Table 3). | 17 &19 |
| Risk of bias across studies | 22 | Except for female gender, the meta-analysis results were very heterogeneous and therefore, a random effect meta-analysis was done. The random effect analysis was also heterogeneous. To sort out the cause, publication bias was assessed. The funnel plot figures in S2A-E figures shows the presence of possible publication bias. This bias might be due to missing of grey literature across the continent and exclusion of non-English language written article. The other causes of heterogeneity might be due to differences in the recruitment criteria of TBLN cases and measurement of outcome variables. Sensitivity analysis was done for gender, age and TBLN/HIV co-infection for which over ten articles had been included. While omission of a single study at a time had minimal influence on the pooled prevalence of TBLN/HIV co-infection, it showed an influence on pooled prevalence of age groups [29, 45] and gender [45]. These two articles have good methodological and outcome data quality. Thus, it is less likely to influence the result away from the true pooled estimate. The GRADE pro system of grading the quality of evidence showed low quality of evidence. This is due to the methodological quality of the articles included in the review. | 18 |
| Additional analysis | 23 | NA |  |
| **DISCUSSION** | | |  |
| Summary of evidence | 24 | Tuberculous Lymphadenitis (scrofula) has been recognized for thousands of years and remains one of the most common forms of EPTB [63]. Cervical TBLN is the most frequent form followed by axillary and inguinal TBLN. In the middle ages in Europe, it was believed that a touch from royalty could heal this disease [64]. Unlike PTB which is more common among males [5], our review identified a relatively higher prevalence of female, 53% among TBLN than male (low quality of evidence) (Fig4A, Table 3). The link between female gender and TBLN is not well known. However, reports showed that difference in tumor necrosis factor, interleukin-10 productions, CD4+ lymphocyte counts, endocrine, socioeconomic and cultural factors [64] might be responsible. A review of 31 articles from Afghanistan, Pakistan, India and Bangladesh agreed with our report [18]. According to this historic paper, pregnancy, diabetes, vitamin D deficiency and low protein consumption [18] were discussed as potential factors associated with TBLN.  The pooled prevalence for the age group of 15-44 years was higher than for other age groups among TBLN cases (low quality of evidence). TBLN was previously considered a disease of childhood [65]. Nowadays, it has a peak age of onset of 20 to 40 years [9, 64]. A critical review by Biadglegn *et al.* (2013) showed that EPTB (with TBLN being the most common presentation) was more common among young adults [17].  Sub-group analysis of TBLN/HIV co-infection by country/sub-region/ showed that the pooled prevalence of HIV among TBLN in African countries other than Ethiopia was 73% whereas it was 21% in Ethiopia (low quality of evidence). This indicates that Ethiopia’s high TBLN rate is probably unique in its epidemiology and seems to lack directional correlation with HIV. Multiple studies showed that, HIV infection was significantly associated with allopatric than sympatric host- pathogen relationships [66-68]. Absence of directional correlation between TBLN and HIV might indicate that both MTBC and its hosts are co-evolved together for long period of time in Ethiopia. Moreover, evidence shows that EPTB is associated with HIV when it has pulmonary extension rather than when it is exclusively localized [9]. The TBLN cases reviewed here are isolated TBLN cases as disseminated TB cases were an exclusion criterion. When considering Africa, the epidemiology of HIV among TBLN appears to be in line with other parts of the world in which HIV is the main driver of EPTB, including TBLN [69].  The majority (88%) of TBLN cases were newly identified cases arguing against an association between TBLN and TB treatment history. The history of eating raw meat/drinking raw milk among TBLN cases in Africa was 46% (low quality of evidence). Historically, it has been reported that 10-20% of all TBLN in Europe was caused by *M. bovis*, acquired from drinking unpasteurized milk [70]. However, recent studies from countries with similar settings (endemic bovine TB in cattle and no pasteurization) [71] have not mirrored such high prevalence of zoonotic TB. For instance, molecular analysis of 173 isolates from pastoral communities who had contact with livestock resulted in 160 *M.tuberculosis* and three *M. bovis*. Similarly, molecular analysis of 39 isolates from their camel, cattle and goat showed 24 *M. bovis* and 1 *M. tuberculosis* [72]. These data confirmed the low incidence of *M. bovis* in human TB. In contrast, a systematic review of global epidemiology of TB due to *M. bovis* showed a high rate, 2.8% of *M. bovis* among human in Africa [73]. However, Müller *et al.* (2013) included articles which identify *M. bovis* based on biochemical methods; which is non-specific for identification of *M. bovis.* Taken together, *M. bovis* rarely detected in human TB [29, 74].  The prevalence of BCG vaccination history among TBLN cases in this review was 24%. However, the number of articles were small (only 4 articles). Thus, the quality of the evidence is low; pending further investigation. There is little data about effects of BCG on the incidence of TBLN, except reports of adverse effects among infants [75].  The prevalence of the cardinal TB symptoms among TBLN cases in Africa ranged from 32% with history of cough for longer than two weeks to 62% with record of weight loss. Overall, the prevalence of one or more systemic symptoms were 49%. Based on this report history of cough was a less prevalent systemic symptom than weight loss. Unlike this study, a study from India showed that, fever was the most prevalent symptom in TBLN [76]. Moreover, the prevalence of one or more systemic symptoms were 56.6% which is slightly higher than this report,49% [76]. Another report from Turkey showed a prevalence of cough of 26-33% which in line with this report and night sweating of 29-36% [77] which is lower compared with the present study, 55%. Similarly, | 20-22 |
| Limitations | 25 | Due to methodological exclusion of articles published in languages other than English and missing of grey literature; publication bias is likely high. Although we included a large number of articles in the review; each article however contained only few variables. Thus, prevalence estimate was based on few articles which might make our pooled estimate imprecise. In addition, most of the included articles were chart reviews and retrospective in nature likely introducing clinical and methodological heterogeneity. These collectively reduce the quality of the generated evidence. | 22-23 |
| Conclusions | 26 | This review is the first comprehensive meta-analysis that estimated pooled prevalence for key demographic, exposure and clinical variables that could characterize TBLN. Of the total 28 articles included in the review, 19 were from the horn of Africa with most of these from Ethiopia (14 studies) suggesting clustering of TBLN in Eastern Africa than in other sub regions. Within Ethiopia, TBLN was also relatively more clustered in agrarian than in pastoral regions.  Most TBLN (77%) were cervical in type, matted (67%) and mobile (64%) in their feature. The majority (68%) were in the age-range of 15-44 years. Unlike PTB which is more prevalent among male; TBLN is slightly higher among women,53% which require further investigation. The TBLN/HIV co-infection rate was 52% for Africa but 21% in Ethiopia and 73% in the rest of African counties which indicates the unique feature of TBLN epidemiology in Ethiopia. Eighty-eight percent of TBLN cases had no prior TB treatment history, 52% had livestock exposure and 24% had BCG vaccine scar. The cardinal systemic symptoms of TBLN showed prevalence of 45%, 55%, 62% and 32% for fever, night sweating, weight loss and history of cough for longer than two weeks, respectively.  To identify the most informative risk factors, a meta-analysis and/or prospective double population-based study is highly desirable. Moreover, the host and pathogen genomic dimension and their evolutionary relationship should be investigated. | 23 |
| **FUNDING** | | |  |
| Funding | 27 | This research received no specific grant from any funding agency | 25 |
